# Phenomena of the Capillary Circulation

**Published:** 1858-09

**Authors:** Austin Flint

**Affiliations:** Buffalo, New York


ART. II.—*Phenomena of the Capillary Circulation.* By AUSTIN FLINT, JR., M.D., of Buffalo, New York.

SINCE it has become the almost universally received doctrine that the vital processes of nutrition, and that most important modification of these processes, namely, inflammation, go on in the capillary system, and since we have been enabled, by the aid of the perfect microscopes which are now in such general use, to bring the phenomena of the capillary circulation immediately under our observation, this subject has acquired additional importance, and, with its physiological and pathological bearings, has met with much careful study and investigation. This was made the subject of an inaugural thesis at the Jefferson Medical College, 1857.* In the preparation of that paper, I emyloyed observations which I had made during the previous summer, nine of which (in the capillary circulation in the web of the frog) I carefully recorded. In making these observations, I found it most convenient to break up the medulla oblongata with a dissecting needle before attempting to view the circulation, as this rendered the frog perfectly manageable, and as it had no effect on the circulation, as shown by the experiments of M. Brown-Séquard, and as confirmed by myself. In addition to these experiments on the web, I observed the circulation in all other parts of the frog where it can be exhibited, especially in the lungs.† The appearances of the circulation in this situation are extremely beautiful; we can distinctly see the air-cells, surrounded by capillary vessels, through which the blood bounds with inconceivable rapidity. It is said by Dr. Willis, and confirmed by Wagner and Gluge, that the transparent plasma which we find occupying the space next the walls of the vessels in most situations, while the blood-disks occupy the centre, constituting the "still layer" of Kirkes, is not observed in this situation; in other words, the vessels are crowded to their very walls with corpuscles. For this remarkable deviation from a general law they offer no explanation.

I have never observed this peculiarity, as my attention was not directed to it while examining the pulmonary circulation. Those who believe that the heart exercises the sole power in the production of the circulation, would be entirely unable to account for this phenomenon; but holding as I do (and I shall more fully explain further on) that there is another very important agent in the production of circulation, namely, an attractive force resident in the tissues, which acts upon the blood, and which is al-

---

* The American Journal of Medical Sciences, July, 1857.

† For description of process of exhibiting the circulation in the lungs, see Art. in Am. Journal.

lied to, or dependent upon, the vital processes of nutrition, it seems to me that it can be explained in the following manner: The blood circulating in the systemic capillaries nourishes the tissues by the liquor sanguinis, and then the attractive force acts upon this constituent, creating a tendency for the plasma to take its position next the walls of the vessels, thus forming the "still layer." But the pulmonary capillary circulation is for the *aeration* of the blood, a process which is effected in the globules, and not in the plasma; the great mass of blood is sent to the lungs for the purpose of aeration, not of nutrition; *hence the globules, which here feel the force of attraction for oxygen, occupy the space next the walls of the vessels.* Taking the view which I do of the causes of the capillary circulation, this explanation seems satisfactory.

The appearances of the capillary circulation in the web of the frog's foot may be described in the following manner:—

When the web is subjected to examination, we have vessels of various sizes in the field, consisting of arterioles and venules, which are exceedingly variable in diameter, and the true capillaries, which are all of nearly equal diameters. The blood is seen coursing along the vessels with great rapidity, especially in the arterioles, where we may observe a slight pulsatory movement.

In the arterioles, as a general rule, the blood moves with unvarying rapidity, and here especially we notice a space next the walls of the vessels, which is not occupied by the red globules, but along which the colorless globules move at a diminished rate, appearing to have a tendency to adhere to the walls of the vessels, and sometimes even remaining stationary for a time, until they are pushed along again by the central mass. This space constitutes the still layer of Dr. Kirkes. The white or colorless corpuscles are much fewer than the red, and move at least ten or twelve times more slowly than the central mass. On careful examination, I have been able to remark a decided difference between the circulation in the arterioles and the venules. In the latter the movement is not so rapid, the globules appearing to be impelled more by a "*vis a tergo*," and to feel less the influence of the "*vis a fronte*," which seems to operate in the arterioles. The comparative number of the white corpuscles is greater, but the "still layer" appears to occupy a smaller proportion of the calibre of the vessel.

In the true capillaries the movements are less regular, and are apparently dependent, in a great measure, upon a force which acts directly upon them; the "capillary power," as it has been designated by Dr. Carpenter. This matter will be more fully touched upon presently, when we consider the causes of the capillary circulation. In the true capillaries, also, the blood moves in every possible direction, at different rates of speed, in different vessels, and also at different times in the same vessel. In one instance, I remarked a capillary branching from a vessel at an acute angle, (that is, turning almost directly opposite to the current in the main vessel,) with individual globules shooting through it with great rapidity. In many instances I have observed an entire stasis in one or two of the capillary vessels—existing, however, only for a moment, the circulation then recommencing with its original vigor. Dr. Carpenter has remarked a stasis followed by a current in an opposite direction.

It frequeutly happens that a globule is caught at the point of junction of two vessels, and remains stationary until carried along by the current of blood. Globules are frequently bent upon themselves as they pass from one vessel to another, but as soon as the cause is removed, they regain their original conformation. The walls of the vessel are motionless, and take no active part in the normal circulation, as was supposed by some of the older writers.

I have now given a description of the capillary circulation, as it appeared to me under the most favorable circumstances; more minute, perhaps, but not otherwise differing from the ordinary descriptions in works on physiology: and then naturally follows a consideration of the causes of the capillary circulation. I say *causes*, because I shall take the ground that it is not produced by a single cause, the heart's contraction, as was supposed by the great discoverer of the circulation. While it may be that the action of the heart is sufficient to propel the blood through the whole round of the circulation, as is contended by Magendie, by Dr. Allen Thompson, in the *Cyclopædia of Anatomy and Physiology*, by Dr. Kirkes, and others, I believe that there are other causes which operate, and which are able to carry on the function of circulation unassisted, as is shown by the acardiac fœtus of Dr. Houston, in a case reported in the *Dublin Medical Journal*, 1837, when, of course, the circulation was stopped at the birth of the child by the want of due aeration of the blood.*

What causes seem to operate in the production of the capillary circulation, judging merely from the appearances under the microscope? In the observations which I have recorded on this point, I have noted an irregularity of movement in the capillaries, both in different vessels at the same time, and in the same vessels at different times; the irregularity sometimes amounting to entire cessation of the circulation in a single vessel, followed by a current in an opposite direction; the shooting off of glo-

* In the American Journal, July, 1857, I went into a discussion of the views of some of our most eminent physiologists on this subject. In this article I shall confine myself merely to a statement of my own convictions, without discussing the opposed opinions.

bules through vessels which were before empty; the darting off of globules through capillary branches with a velocity greater than that of the blood in the main vessel; and, in short, all the phenomena which are presented to the eye seem to indicate that there is an attractive force resident in the solid vital particles which operates on the blood in the capillaries.

We are not supposing the existence of a force, with the operation of which we are unacquainted. The present school of physiology teaches us that the processes of nutrition, of molecular disintegration, and of secretion, are dependent upon a vital force resident in the solid particles of the organism, which are essentially vitalized; and inflammation is now supposed to be due to a perversion of this force. In what other way could we explain the fact that every tissue takes from the mass of the arterial blood the substances which are required for its nutrition? The blood sent to the systemic capillaries by the heart is the same in all parts of the body; but when the great change which is effected in these vessels takes place, we find that the blood, which has thus been rendered venous, has not the same chemical constitution or the same appearance in all veins; for example, the blood of the emulgent vein is almost as florid as arterial blood.

The existence of a distinct capillary action is now believed by the highest authorities. Lehmann believes that a chemico-vital attraction of the blood for the tissue, together with the physical capillary attraction, produces the movement of the blood in the capillaries, and forces it into the veins. Dr. Carpenter believes that there exists a "capillary power" which is superadded to the force of the heart. Professor Dunglison teaches that there is an independent power resident in the tissues about the capillaries, and that, "by the united action of the heart, arteries, and capillaries, or intermediate system of vessels, the blood attains the veins." Even those who recognize the heart as the only efficient organ of the circulation, yield that the capillaries possess a "distributive force;" that is, though the circulation is effected by the unassisted action of the heart, yet the tissues have an attraction or affinity for the blood, which distributes it for the nutrition to each and every part of the body.

Taking into consideration everything which I have seen bearing on this subject, it seems to me to be clearly proved that the normal capillary circulation is dependent mainly upon the action of the heart and the "capillary power," or attractive force resident in the vital particles. It cannot be denied that the heart has a considerable share of the duty of capillary circulation. Taking into account the condition of the blood and vessels, apparently a slight force is capable of propelling the blood through the capillary system. When a small artery is divided, the force with which the blood is expelled is considerable, and appears sufficient to exert a decided effect upon the motion of the blood in the capillary system. It is impossible to estimate with much accuracy the proportional influence which the heart exerts in producing capillary circulation. The vital affinity between the tissues and the blood, which I suppose to be the other power concerned in this function, never ceases; still, as the action of the heart is often much interfered with, as in cases of excessive fatty degeneration, and as the heart has been removed from the frog, the capillary circulation nevertheless continuing, we cannot think that its power is greater than the attractive force, which I hold to be essentially concerned in the performance of that function. The extent of the heart's action is also variable, both in different individuals and in the same individual at different times.

The only other force which has any share in the production of capillary circulation, unless it be a slight suction force from the veins, is the "capillary power." This seems to me to play the more constant and effective part; when this ceases to act, the animal dies, and the blood refuses to circulate in spite of the efforts of the heart. This is the great vital force of nutrition which is constantly operating, and which is so wonderful and inexplicable. We know that there is such a force, and that it continually acts, but what it is, or what is its essential character, is beyond the wisdom of man to explain It is the life which was breathed into man at his creation, and which is as wonderful and inexplicable now as ever.

Lastly, the following inquiry suggests itself:—What conditions are necessary to the healthy performance of the capillary circulation?

We can answer; in the first place, a healthy condition of the vital particles, which is produced by healthy nutrition; and, in the second place, a particular condition of the blood, which is effected by respiration.

No arguments appear to be necessary to prove the first statement, but I have made experiments, which I shall proceed to describe, which conclusively establish the second point.

The following experiment, made by Dr. J. Reid, and reported in the *Edinburgh Med. and Surg. Journal*, April, 1841, is quoted by Dr. Carpenter:—

Dr. Reid found that when the ingress of air through the trachea of a dog was prevented, and the asphyxia was proceeding to the stage of insensibility, the pressure in the femoral artery, indicated by the hæmadynamometer, was much greater than usual. Upon applying a similar test to a vein, the pressure was proportionally diminished, whence it became apparent that there was an unusual obstruction to the passage of the venous blood (the blood being venous in the arteries) in the systemic capillaries.

Before seeing an account of this experiment, I made the following observations with reference to the same point, carefully recording them :—

*Observation First.*—The medulla of a middle-sized frog was broken up, and the web submitted to microscopic examination. The frog was bathed in sulphuric ether, (care being taken to allow none to touch the web under examination,) and the circulation watched for ten minutes No effect could be discovered. The object of this experiment was to determine whether the phenomena in the succeeding experiment were in any degree dependent upon the other, which was conducted in collodion. The frog was then painted over with an impermeable coating of collodion, care being taken, as before, not to touch the web. The effect on the circulation was immediate. It instantly became less rapid, until, at the expiration of twenty minutes, it had entirely ceased. The smaller vessels were the first to become affected, the larger arterials resisting its action longest. One of the first effects was a pulsative movement in the vessels where the blood had previously flowed in a continuous stream, showing *that the attractive force is lost, but the heart's action is still felt.* The fact of the first arrest of the blood in the capillaries, seems to indicate that it is now unfit to supply the wants of the tissues, and therefore the attractive force ceases to be operative. The arrest of the circulation was steady, and at the expiration of twenty minutes the motion had entirely ceased. The entire coating of collodion was now instantly peeled off, and the effect on the circulation was instantaneous. Quite a rapid circulation immediately commenced, but it soon began to decline, and in twenty minutes had almost ceased. The heart was now exposed, and found contracting regularly. In making this experiment, *all respiration* was abolished; the breaking up of the medulla oblongata destroying the pulmonary, and the impervious coating, applied to the entire surface, destroying the cutaneous respiration.

*Observation Second.*—I painted a frog with a thick coating of collodion without breaking up the medulla. It struggled vigorously at first, but soon became quiet, and the web was put under the microscope.

The circulation was affected in the same manner as in the preceding observation, and entirely ceased in twenty-five minutes. During the first few minutes the nostrils dilated and contracted rapidly, but soon became motionless, care having been taken not to obstruct them with collodion, though it was applied effectually to all other parts excepting the web under observation.

The experiment of Dr. Reid proves the fact, inferentially, that blood deprived of oxygen, as in asphyxia, is retarded in the systemic capillaries; but the experiments just related bring the processes directly under the eye, and we can see clearly that when the blood is not aerated, though the heart continues to contract, it will not circulate, and that the situation in which it is retarded is in the capillary system. My second experiment demonstrated the comparatively small part which the lungs of the frog take in respiration; the blood circulating in the frog in which the cutaneous respiration alone was abolished, and the pulmonary respiration not interfered with, but five minutes longer than in the experiment, when both kinds of respiration were abolished. Capillary circulation will go on after tying the trachea, the blood being sufficiently aerated by means of the skin.

Thus it is experimentally proved that an oxygenated state of the blood is an indispensable condition for its circulation through the capillaries; and when the process of respiration or aeration of the blood is abolished, the blood cannot circulate. This we know to be the fact, but we demonstrate by the preceding experiments that in asphyxia the impediment to the circulation exists in the capillaries; that the condition of oxygenation is necessary to the performance of the vital functions, and, it may be, that the entire want of capillary power throws all the onus on the heart, which organ is insufficient for the labor thus imposed upon it. In one of my experiments, after the capillary circulation had entirely ceased, the thorax was opened, and the heart found beating regularly.

